# Coupling coordination and underlying mechanisms of urbanization development and land use efficiency in the Gansu section of the Yellow River Basin

**DOI:** 10.1371/journal.pone.0301784

**Published:** 2024-04-05

**Authors:** Chenyu Lu, Tong Zhang, Wendi Zhang

**Affiliations:** 1 School of Architecture and Urban Planning, Lanzhou Jiaotong University, Lanzhou, China; 2 College of Geography and Environmental Science, Northwest Normal University, Lanzhou, China; Qufu Normal University, CHINA

## Abstract

This swift progression of urbanization has led to increasingly prominent conflicts over the use of land, particularly around its supply and demand. Researchers, both in China and internationally, have underscored the inherent interconnection between urbanization and land utilization. This relationship has gradually become more complex with the development of urbanization. With the implementation of the Yellow River Basin’s strategy to preserve the environment while ensuring high-quality development, the Yellow River Basin has become a focal point of attention for numerous scholars. This study centers on the 57 county-level administrative divisions within the Gansu segment of the Yellow River Basin. We employed an extensive array of methodologies, such as GIS technology, the entropy method, data envelopment analysis, the coupling coordination degree model, and the panel vector autoregressive model. We established an index system and a measurement model to evaluate the degree of urbanization and the efficiency of land use. We also investigated the coupling coordinated dynamics between these two variables, to further explore the dynamic interplay between urbanization and land use and reveal their underlying mechanisms. The conclusions are as follows. The urbanization level and efficiency of land use in the Gansu section of the Yellow River Basin have exhibited a consistent upward trajectory, albeit at levels that are not particularly high, indicating substantial room for improvement in the future. The level of coupling coordination between urbanization and land use efficiency in the Gansu section of the Yellow River Basin has shown a generally upward trend. However, the overall coordination level remains relatively low, characterized by an imbalance, with “high coupling but low coordination”. Regarding spatial distribution patterns, considerable disparities exist in the level of coordination development, which generally decreases from the eastern toward the western regions. A strong reciprocal and interactive relationship exists between the urbanization level and land use efficiency. An elevated level of economic urbanization can initially stimulate land use efficiency. Similarly, the improvement in the level of population urbanization, social urbanization, and ecological urbanization tends to exert a restraining influence on the augmentation of land use efficiency. Conversely, the enhancement of land use efficiency makes a distinct contribution to promoting the elevation of the urbanization level.

## 1 Introduction

Urbanization is imperative for social and economic development, serving as an indispensable route to attain socialist modernization [1, 2]. Since the 1980s, China has embarked on a period of swift growth in urbanization [3]. Between 1978 and 2021, China’s urbanization rate jumped from 17.92% to 64.72%; the average annual growth rate was 3.03% [4]. This swift progression of urbanization has led to increasingly prominent conflicts over the use of land, particularly around its supply and demand [5]. New urbanization is further deepened by the conceptual connotation of urbanization, the core of which is people-oriented, with environmental protection as the prerequisite, and the principle of integrating and balancing, to promote urban modernization, urban ecology, rural urbanization, and to improve the quality and overall level of urbanization [6]. The “National New Urbanization Plan 2014–2020”, which was published in 2014, underscored the importance of optimizing land use structure and enhancing land use efficiency as pivotal objectives in the context of new urbanization development. In 2022, the “14th Five-Year Plan for New Urbanization Implementation” reiterated the importance of bolstering spatial governance and increasing the intensity and efficiency of land-resource use. As a pivotal catalyst for social and economic development, advances in the urbanization processes have fundamentally reshaped the structure and configuration of land utilization. Moreover, land use changes have also exhibited clear spatial dependencies in relation to urbanization [7]. Researchers, both in China and internationally, have conducted comprehensive studies of the interplay between urbanization and land use, and all have underscored the inherent interconnection between urbanization and land utilization [8, 9]. This relationship has gradually become more complex with the development of urbanization [10–13].

**Fig 1:**
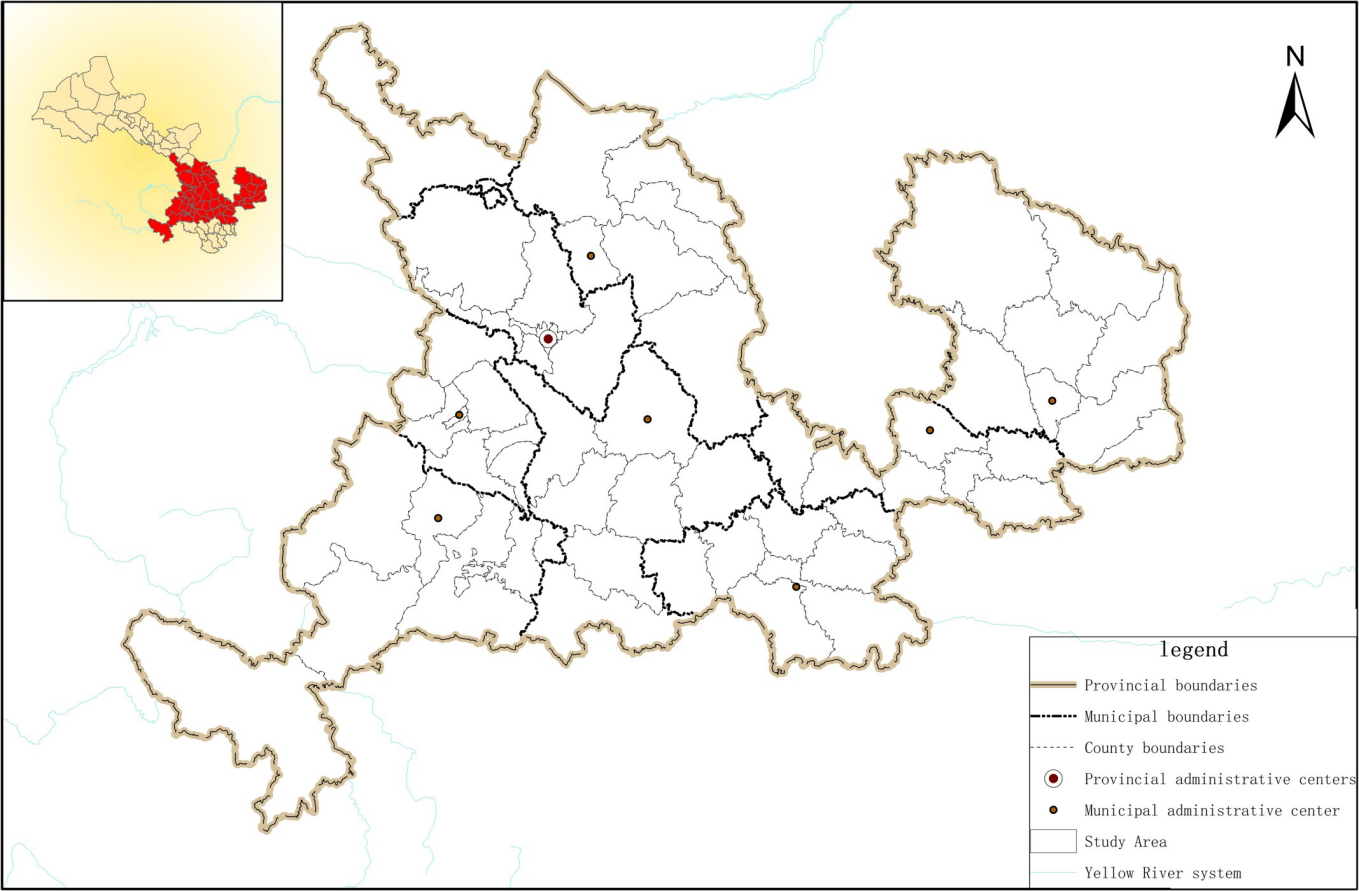
The study area’s geographic boundaries.

Since the commencement of the 14th Five-Year Plan period, with the implementation of the Yellow River Basin’s strategy to preserve the environment while ensuring high-quality development, the Yellow River Basin has become a focal point of attention for numerous scholars, who have engaged in extensive research. The field of urban and rural development has emerged as a pivotal battleground in the pursuit of ecological preservation alongside the high-quality development being undertaken in the Yellow River Basin [14]. The total area of Gansu section of the Yellow River Basin is 145,900 square kilometers, with grassland, cultivated land, and forest land as the main types of land use, accounting for 53%, 30%, and 12% respectively. Located in the transitional zone of China’s three major natural geographical regions, the misalignment of water and soil resource allocation results in lower land use efficiency than in coastal open cities. Influenced by the level of economic and social development and geographical location, the low level of urbanization and industrialization, coupled with prominent contradictions in land use, pose numerous challenges including resource and environmental constraints, fragile ecosystems, and low urbanization rates. In 2019, the rate of urbanization rate in this region lagged behind the national average by 3.7%, with Gansu Province notably far behind, at approximately 10% below the national average [15]. The Gansu segment of the Yellow River Basin is located in the upper and middle sections of the Yellow River, in the east of Gansu Province (Fig 1). It comprises 57 county-level administrative divisions, accounting for 19% of the total land area of the Yellow River Basin. There is relatively limited economic capacity here, with marked disparities in urban and rural development, underdeveloped infrastructure, varied geographical features, and an overall fragile ecological environment [16, 17]. In the new era context, there is still great demand for land resources due to social and economic development. The Yellow River enters and exits Gansu province at two distinct points, reinforcing the pivotal role it plays in protecting the environment of the Yellow River Basin while simultaneously continuing with high-quality development in the region.

This study centers on the 57 county-level administrative divisions within the Gansu segment of the Yellow River Basin. We employed an extensive array of methodologies, such as GIS technology, the entropy method, data envelopment analysis, the coupling coordination degree model, and the panel vector autoregressive model. We established an index system and a measurement model to evaluate the degree of urbanization and the efficiency of land use. We also investigated the coupling coordinated dynamics between these two variables, to further explore the dynamic interplay between urbanization and land use and reveal their underlying mechanisms. This research has substantial theoretical and practical importance. It augments and strengthens the research framework for urbanization and land use, both theoretically and empirically, and enriches sustainable development and human geography theory. It also provides theoretical support and decision-making guidance to achieve development within the Gansu segment of the Yellow River Basin that is of high quality.

## 2 Literature review

In relation to the influence that the process of urbanization has on land use efficiency, some international researchers hold that urbanization plays a substantial role in improving land use efficiency. For example, Defries and colleagues suggested that urbanization has a positive, promoting effect on enhancing land use efficiency [18]. Friedmann argued that an increase in urbanization levels triggers ongoing adjustments in the industrial structure, ultimately leading to improved land use efficiency [19]. Wieand suggested that an increase in urbanization levels leads to a more efficient utilization of land as a primary production factor [20]. Carliner contended that an increase in urbanization levels drives the enhancement of land’s economic benefits [21]. Marco and colleagues proposed that disparities in the level of urbanization development result in diverse urban functions, consequently influencing land use efficiency [22]. Nevertheless, there are researchers who hold contrary opinions. For example, Braid and colleagues argued that the urbanization process may diminish land use efficiency [23]. Freeman suggested that urban spatial expansion can also result in a decrease in the efficiency of land use [24].

Regarding the influence of land use efficiency on the urbanization process, scholars have undertaken research from diverse perspectives. In the late 20th century, American researchers introduced the concept of “smart growth” as a model for urban development, emphasizing the control of urban sprawl by enhancing land use efficiency [25]. Research conducted by Shen and colleagues suggested that, under government guidance, enhancing land use efficiency is beneficial for urbanization and sustainable development [26]. Ullah and colleagues conducted an assessment of land suitability in Dhaka, Bangladesh, and provided land use proportions for various land categories during the course of urbanization development [27]. Copeland and colleagues argued that enhancing land use efficiency represents a critical pathway to elevating the level of urbanization development [28]. Kalfas and colleagues believed that land resources are a key factor constraining the social and economic development [29].

Researchers have conducted a series of studies to investigate the urbanization process and its relationship with land use efficiency. Louw’s research suggested that land use efficiency and urbanization exhibited a strong correlation [30]. Hui and colleagues discovered that complex interrelationships exist between urbanization and the efficiency of land use; these interrelationships can be either positive or negative [31]. Some researchers have approached this issue from the perspective of coupling coordination and interaction mechanisms. For example, Chen analyzed the urban compactness and urban land-use efficiency, as well as the degree and types of coupling and coordination between them [32]. Xiao and colleagues explored the coupling coordination mechanism between land use and urban development; their focus was the Loess Plateau region of China [33]. Some researchers have employed panel vector autoregressive (PVAR) models to examine the mechanisms of interactions between urbanization and land use. For instance, Zheng and colleagues conducted research into the dynamic mechanisms of reciprocal influence between urbanization and land use, using impulse response and variance decomposition methods [34]. Zhao and colleagues employed impulse response and stationarity testing techniques to explore interactions between the degree of urbanization and land use in three urban clusters: the Beijing-Tianjin-Hebei region, the Pearl River Delta, and the Yangtze River Delta [35].

In general, important progress has been made in this field of research. However, some limitations persist. First, systematic and comprehensive research into coupling coordination, dynamic responses, and the mechanisms of interactions between urbanization and land use remain at an exploratory stage, with a relative scarcity of findings. Second, in terms of the scale of existing research, most studies in this field have focused on relatively large spatial scales, such as urban clusters, provinces, and cities, with limited exploration at the county level. There is thus a relative shortage of research results based on the county-level scale. In this study we aimed to address these deficiencies and shortcomings.

## 3 Data and methods

### 3.1 The index system and data sources

Adhering to the principles of scientific rigor, hierarchy, and selectivity, and taking into account the specific conditions and characteristics of the study area, we have developed an index system to assess the degree of urbanization and the efficiency of land use in the Gansu segment of the Yellow River Basin (Table 1). The index system for assessing the degree of urbanization has been constructed using four dimensions: population, economy, society, and ecology. This framework is grounded in theoretical discussions regarding the essence of new urbanization [3, 11, 36] and references the “evaluation index system for in situ urbanization” [37]. For the index system for evaluating land use efficiency, input-related indicators are selected from capital input, labor input, and land input, while output-related indicators are chosen from economic benefits, social benefits, and environmental benefits [36, 38, 39].

**Table 1:** Index system to evaluate urbanization development and land-use efficiency.

	Criterion	First-level indicators	Second-level indicators
**Evaluation index system for urbanization development**	Population urbanization	Proportion of urban population	
		Density of urban population	
		Proportion of non-agricultural employment	
	Economic urbanization	Per capita GDP	
		Savings deposited by urban and rural residents	
		Proportion of non-agricultural industries	
	Social urbanization	Level of education	Number of full-time secondary school teachers / number of secondary school students
		Medical and health conditions	Number of medical and health facility beds / total population
		Communication facility level	Number of landline telephone subscribers + number of mobile phone subscribers + number of internet broadband access users) / total population
		Transportation facility level	Total road network size / total area of county
	Ecological urbanization	Urban greening coverage rate	
		Harmless treatment rate of urban household waste	
		Urban wastewater centralized treatment rate	
**Evaluation index system for land-use efficiency**	Input	Capital input	Total fixed assets
		Labor input	Number of individuals employed in secondary/tertiary industry
		Land input	Urban built-up area
	Output	Economic benefits	Value added by secondary/tertiary industry
		Social benefits	Per capita net income
		Environmental benefits	Per capita green area

Our research encompasses the time frame spanning from 2000 to 2020. The primary data sources include annual publications such as the China County Statistical Yearbook, Gansu Development Yearbook, and Gansu Rural Statistical Yearbook. Additionally, data were gathered from various regional statistical reports, national economic/social development statistical bulletins, and other pertinent statistical and literary sources.

### 3.2 Research methods

#### 3.2.1 Entropy method

To assess the level of urbanization development using the entropy method, the specific formula is as follows [40]:

1) Standardize the indicator data:

Positive indicators:

$$z_{ij}=\frac{X_{ij}-X_{\min}}{X_{\max}-X_{\min}}$$ (1)

Negative indicators:

$$z_{ij}=\frac{X_{\max}-X_{ij}}{X_{\max}-X_{\min}}$$ (2)

2) The proportion coefficient of the j-th indicator for county i was calculated:

$$P_{ij}=\frac{X_{ij}}{\sum_{i=1}^{m}X_{ij}}$$ (3)

3) The entropy value of indicator j was calculated:

$$E_{j}=-\frac{1}{\mathrm{lnm}}\sum_{i=1}^{m}P_{ij}lnP_{ij},(j=1,2,\ldots ,n)$$ (4)

4) The coefficient of variation for indicator j was calculated:

$$G_{j}=1‐E_{j}$$ (5)

5) The weight of indicator j was calculated:

$$W_{j}=\frac{G_{j}}{\sum_{j=1}^{n}G_{j}}$$ (6)

6) The comprehensive evaluation value was calculated:

$$C_{i}=\sum_{j=1}^{n}W_{j}X_{ij}$$ (7)

#### 3.2.2 Data envelopment analysis

Land use efficiency is evaluated using the data envelopment analysis (DEA) method. To account for the dynamic nature of the research subject, the variable returns to scale (VRS) output-oriented BCC model was used to calculate the efficiency of land use. The formula used was [41]:

$$\left\{ \begin{array}{c} min\theta \\ s.t.\sum_{j=1}^{n}\lambda_{j}x_{j}+s^{-}=\theta x_{j0}\\ \sum_{j=1}^{n}\lambda_{j}x_{j}-s^{+}=y_{j0}\\ \sum_{j=1}^{n}\lambda_{1}=1\\ \lambda_{j}\geq 0,j=1,2,\cdots ,n\\ s^{+}\geq 0,s^{-}\geq 0 \end{array}\right.$$ (8)

In this equation, θ is employed to denote the measure of relative efficiency. When θ = 1, it signifies the relative efficiency of land use; λ_j_ represents the weight variables; s^+^ and s^−^ represent the slack and surplus variables, respectively, signifying the adjustments in input and output values necessary to achieve the optimal allocation; and x_j0_ and y_j0_ represent the initial inputs and outputs, respectively, of each decision-making unit.

#### 3.2.3 The coupling coordination model

Utilized for quantifying the interactions and reciprocal facilitation among two or more systems, the specific formula is as outlined below [33, 38]:

$$C=2\sqrt{\frac{U_{i}*E_{i}}{{\left(U_{i}*E_{i}\right)}^{2}}}$$ (9)

$$T=\alpha U_{i}+\beta E_{i}$$ (10)

$$D=\sqrt{C*T}$$ (11)

Where U_i_ represents the urbanization development index; E_i_ represents the land use efficiency index; C represents the coupling degree; T represents the comprehensive coordination index; and D represents the degree of coupling coordination. α+β = 1. Recognizing the equal significance of both urbanization and land use, each is assigned a weight of 0.5. Based on the existing research results in the literature about the division standards of coupling coordination degree by relevant scholars [42, 43], and combining the calculation results of the D value, the coupling coordination degree is divided into ten levels (Table 2).

**Table 2:** Classification of the degree of coupling coordination.

Coupling coordination degree	Coordination level	Coordination degree	Coupling coordination degree	Coordination level	Coordination degree
[0.0–0.1]	1	Extremely imbalanced	[0.5–0.6]	6	Barely coordinated
[0.1–0.2]	2	Seriously imbalanced	[0.6–0.7]	7	Slightly coordinated
[0.2–0.3]	3	Moderately imbalanced	[0.7–0.8]	8	Intermediately coordinated
[0.3–0.4]	4	Slightly imbalanced	[0.8–0.9]	9	Well coordinated
[0.4–0.5]	5	Barely imbalanced	[0.9–1.0]	10	Highly coordinated

#### 3.2.4 Panel vector autoregressive (PVAR) model

A PVAR model was constructed to measure the dynamic interaction between the level of urbanization and the efficiency of land use, with the following formula [35]:

$$Y_{it}=\sum_{j=1}^{p}\beta_{j}Y_{it-j}+\eta_{i}+\phi_{t}+\varepsilon_{it}$$ (12)

Where Y_it_ represents the degree of urbanization development and land use efficiency; i represents the number of counties; t represents the study period; p is the lag coefficient; and β_j_ represents the coefficient matrix. Recognizing the regional disparities in the level of urbanization development and the efficiency of land use across counties, we introduced a variable, η_i_, to capture county-specific fixed effects; ϕ_t_ represents distinct impact effects for each time period; and ε_it_ represents the random error term.

## 4 Results and discussion

### 4.1 Spatial and temporal characteristics of urbanization development and land use efficiency

#### 4.1.1 Temporal characteristics

The degree of urbanization and the efficiency of land use in the Gansu segment of the Yellow River Basin were measured using both the entropy method and the data envelopment analysis method (Tables 3 and 4). The findings indicated that both the degree of urbanization development and land use efficiency have exhibited a generally increasing trend over time. However, the overall levels remain relatively modest, indicating considerable potential for improvement in the future. Notably, the increase in the urbanization development level slightly outpaced the increased efficiency of land use.

**Table 3:** The measurement of urbanization development levels.

Year	Maximum value	Minimum value	Range	Standard deviation	Average
2000	0.7746	0.0750	0.6996	0.1056	0.1724
2005	0.7810	0.0780	0.7030	0.1060	0.1808
2010	0.7829	0.0930	0.6899	0.1028	0.1949
2015	0.7803	0.1090	0.6713	0.1010	0.2211
2020	0.7939	0.1127	0.6811	0.1038	0.2418

**Table 4:** The measurement of land use efficiency.

Year	Maximum value	Minimum value	Range	Standard deviation	Average
2000	1.0000	0.1430	0.2801	0.8570	0.5061
2005	1.0000	0.1550	0.2587	0.8450	0.5489
2010	1.0000	0.2290	0.2522	0.7710	0.5779
2015	1.0000	0.2830	0.2344	0.7170	0.5863
2020	1.0000	0.2910	0.2363	0.7090	0.5995

In terms of urbanization development, the Gansu segment of the Yellow River Basin experienced a slower rate of improvement prior to 2010. However, the pace of urbanization development accelerated considerably after 2010, resulting in a rapid increase. Conversely, in the case of land use efficiency, there was a relatively rapid rate of improvement before 2010, but the rate of improvement decelerated after 2010, leading to a comparatively slower increase. The overall range and standard deviation of both the urbanization development and land use efficiency exhibited a fluctuating downward trend, signifying a reduction in regional disparities.

#### 4.1.2 Spatial characteristics

**Fig 2:**
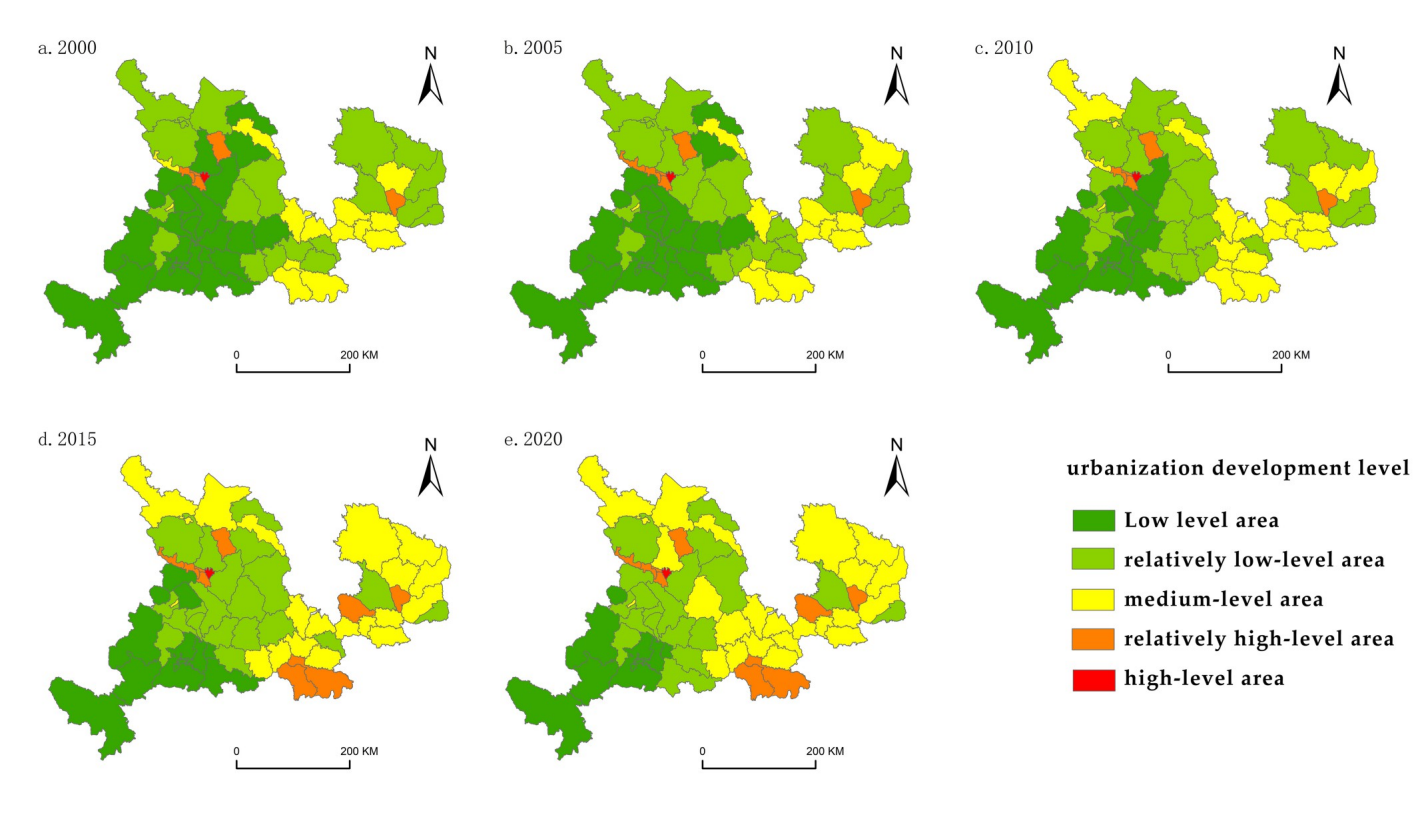
Spatial pattern of urbanization development level.

**Fig 3:**
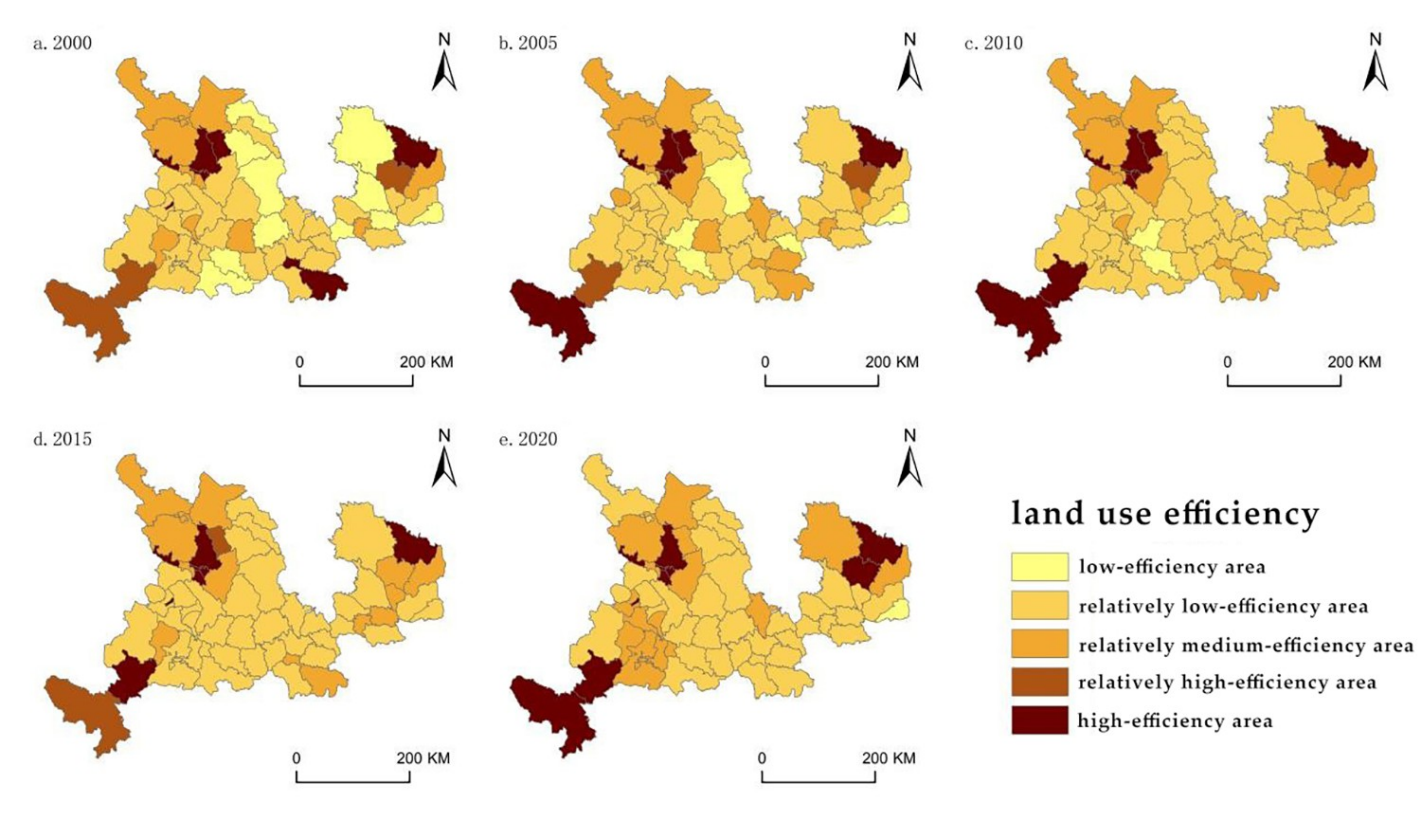
Spatial pattern of land use efficiency.

Utilizing ArcGIS software and employing the natural breakpoints method along with the frequency analysis method, the counties were classified into various tiers of urbanization development based on measurements. These tiers included low-, relatively low-, medium-, relatively high-, and high-level areas (Fig 2). Similarly, based on the measurement outcomes of land use efficiency, the counties were divided into high-, relatively high-, relatively medium-, relatively low-, and low-efficiency areas (Fig 3).

Between 2000 and 2020, there were notable shifts in the urbanization development level, with approximately 56.14% of county-level administrative areas experiencing changes in their classification. The primary transition patterns were from low- to relatively low-level areas and from relatively low- to medium-level areas. Areas classified as high-level and above increased from 10.53% in 2000 to 19.30% in 2020, primarily concentrated in the central counties of Lanzhou and various nearby prefectures, continuously expanding into the surrounding regions throughout the two decades. Conversely, areas classified as low-level and below decreased from 66.66% in 2000 to 42.10% in 2020; these were primarily concentrated in economically underdeveloped regions such as Linxia Prefecture.

Between 2000 and 2020, the overall land use efficiency remained relatively low, with a predominant concentration of relatively low-efficiency areas. High-efficiency areas were primarily observed in specific counties within Lanzhou, characterized by higher urbanization levels, accelerated economic development, relatively well-developed infrastructure, and comparatively high land use efficiency. The count of low-efficiency areas displayed a fluctuating downward trend, initially mainly concentrated in certain counties of Qingyang, Baiyin, and Dingxi cities, with Zhengning County being the only remaining low-efficiency area by 2020.

### 4.2 Temporal and spatial characteristics of coupling coordination development

#### 4.2.1 Temporal characteristics

**Fig 4:**
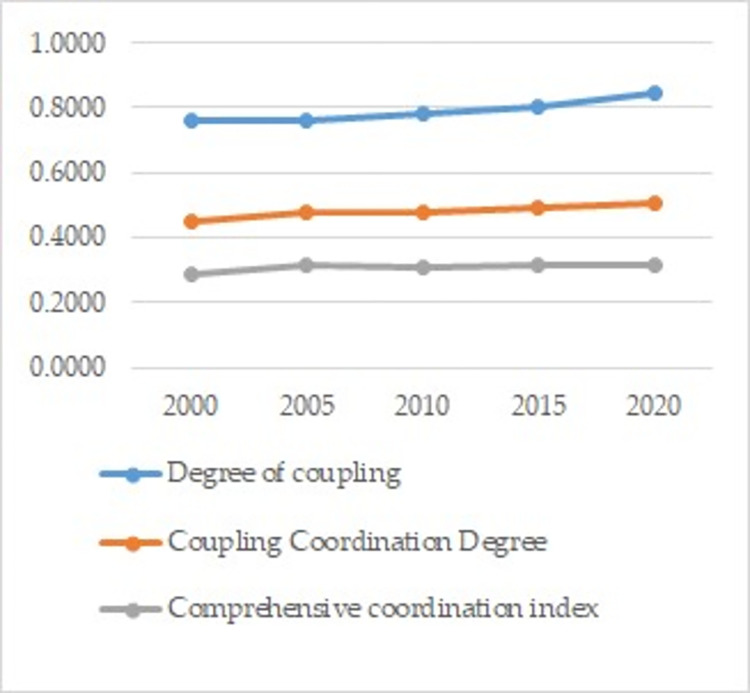
Trends in coupling coordination parameters.

Using the coupling coordination degree model, we quantified the coupling degree, comprehensive coordination index, and coupling coordination degree between the urbanization development and efficiency of land use within the Gansu segment of the Yellow River Basin (Fig 4). In general, all of the parameters of coupling coordination showed an upward trend. As the urbanization process advanced, human activities intensified land use, leading to a gradual increase in the coupling degree between the two systems, indicating a strengthening interaction between urbanization and land use. Although the comprehensive coordination index showed some growth, the overall level remained relatively low. The degree of coupling coordination also increased, transitioning from being nearly uncoordinated into a state of marginal coordination, although the level of coordinated development remained relatively low. The coupling degree between urbanization development and land use efficiency was much higher than the coupling coordination degree, suggesting an imbalanced development pattern characterized by “high coupling but low coordination” features.

#### 4.2.2 Spatial characteristics

**Fig 5:**
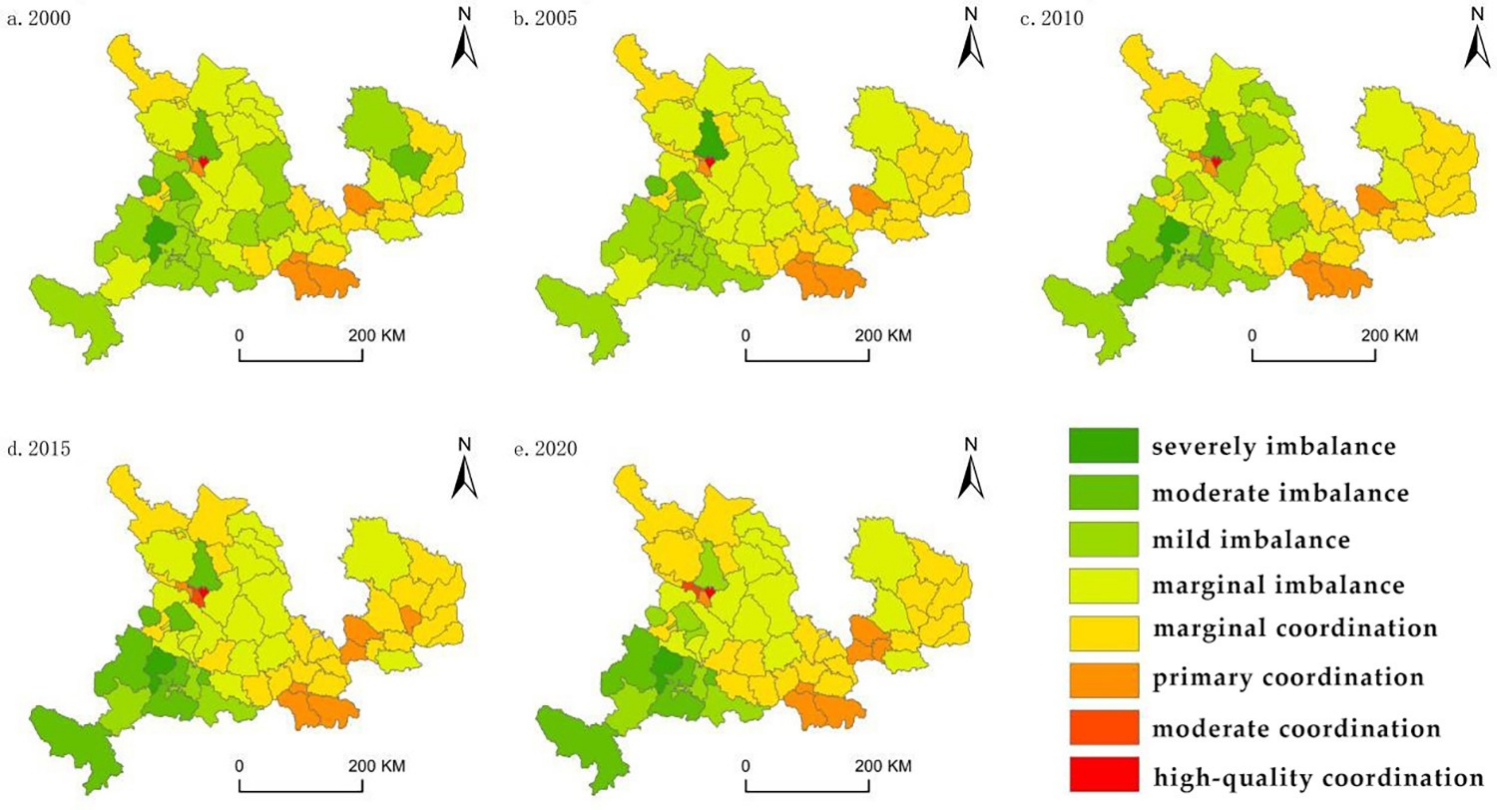
The spatial pattern of coordination development level.

Using our findings regarding the degree of coupling coordination, we categorized and visualized the level of coordinated development between urbanization development and land use efficiency (Fig 5). In general, there were notable spatial differentiation patterns in the degree of coordinated development between urbanization development and land use efficiency, with a gradual decrease in coordination from the eastern toward the western regions. In the eastern region, the relatively high degree of coordinated development has essentially reached the level of marginal coordination or higher. The region around Lanzhou in the central part also exhibits relatively high levels of coordinated development. By contrast, the western regions, including Gannan and Linxia Prefectures, are generally in a state of imbalance.

Specifically, the proportion of areas in a state of marginal imbalance decreased from 35.09% in 2000 to 22.80% in 2020, primarily concentrated in Baiyin and Dingxi cities. Areas in a state of mild imbalance decreased from 24.56% in 2000 to 14.04% in 2020, mainly located in Linxia and Gannan Prefectures. The proportion of areas in a state of moderate imbalance decreased from 7.02% in 2000 to 5.26% in 2020, primarily found in certain counties of Gannan Prefecture. Areas in a state of marginal coordination or higher increased from 21.05% in 2000 to 40.35% in 2020, representing the largest change in coordinated development types, mainly distributed in Qingyang, Tianshui, and Pingliang cities. Areas in a state of primary coordination increased from 8.77% in 2000 to 10.53% in 2020, primarily located in the urban districts of Lanzhou, Tianshui, and Pingliang. By 2020, Chengguan District in Lanzhou was the only region achieving high-quality coordination, while Hezuo City in Gannan Prefecture was the only severely imbalanced area.

### 4.3 Mechanisms of urbanization development and land use efficiency

#### 4.3.1 Model construction

Variables were selected from a range of four urbanization perspectives: population, economic, social, and ecological. We constructed a PVAR model and used it to analyze the mechanisms via which urbanization development and land use efficiency interact (Table 5).

**Table 5:** The selection of variables.

	Variable	Meaning
Urbanization	U1	Population urbanization
	U2	Economic urbanization
	U3	Social urbanization
	U4	Ecological urbanization
Land use efficiency	LE	Land use efficiency

#### 4.3.2 Stationarity tests

The stationarity test (HT) method was chosen to assess the stationarity of the variables, as it is appropriate when conducting unit root tests in short panel data. Additionally, outcomes from the LLC test, ADF test, and PP test were consulted as points of reference. The findings affirmed that all variables successfully passed the stationarity tests, thus enabling further quantitative analysis (Table 6).

**Table 6:** Unit root test results.

Variable	HT	LLC	ADF	PP
U1	-0.34163***	-144.294***	142.789**	213.062***
U2	-0.4284***	-30.7040***	143.791**	238.221***
U3	-0.3403***	-24.2122***	186.001***	292.802***
U4	-0.1730*	-55.6523***	250.524***	362.244***
LE	-0.2406**	-127.220***	147.700***	213.455***

Note: *, **, and *** refer to significance at 10%, 5%, and 1%, respectively.

#### 4.3.3 The optimal lag order

The variables underwent AIC, BIC, and HQIC tests, ultimately confirming that for the PVAR model one lag order was optimal (Table 7).

**Table 7:** AIC, BIC, and HQIC test results.

lag	AIC	BIC	HQIC
1	-33.3278*	-98.7054*	-58.7358*
2	-11.1575	-33.6310	-19.8914

#### 4.3.4 Impulse response analysis

**Fig 6:**
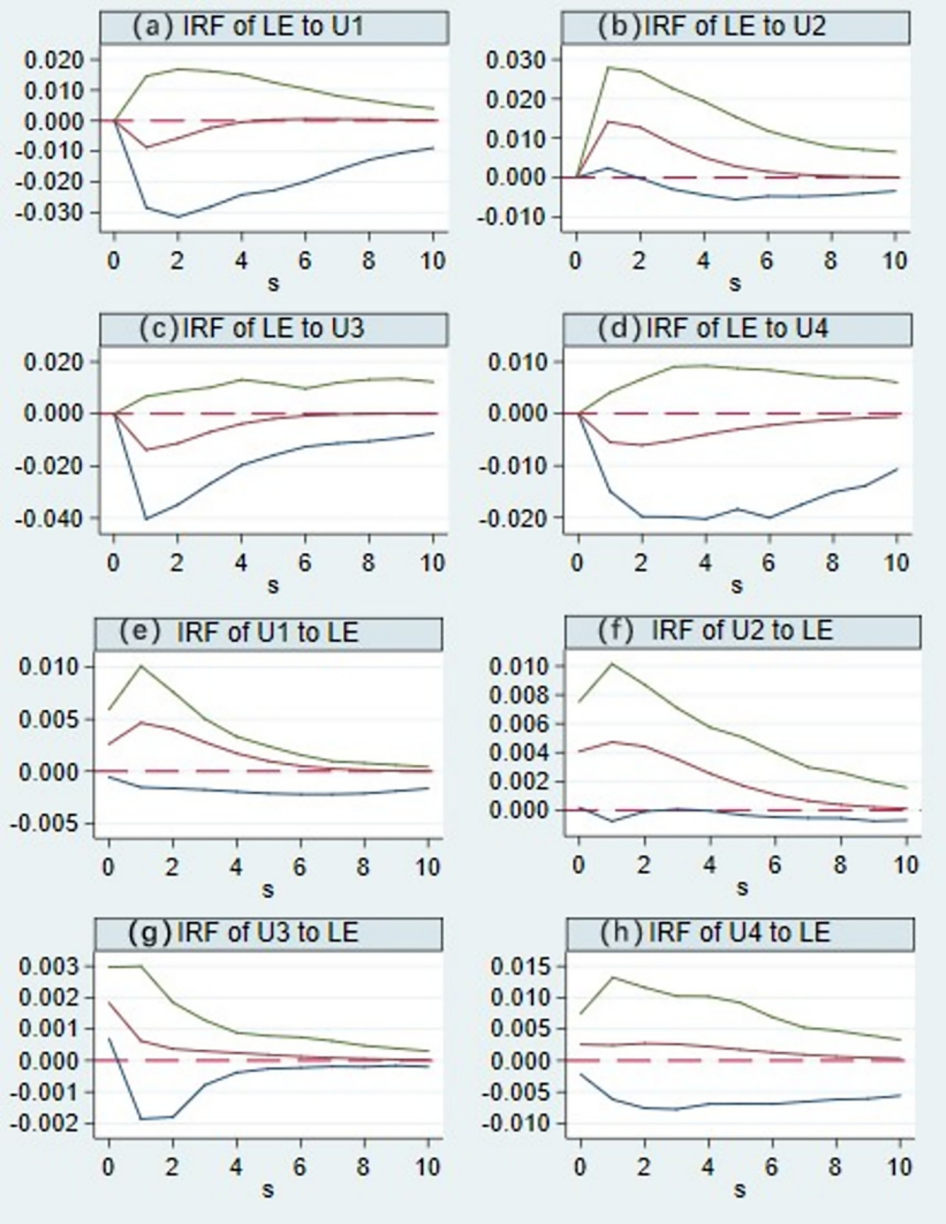
Impulse response curves of urbanization level and land use efficiency.

We performed impulse response analysis with Stata (version 15.0) to examine the relationship that existed between urbanization development and land use efficiency, with the impulse response curves generated (Fig 6). The Impulse Response Function (IRF) can characterize the specific fluctuation trajectory of the independent impact of each endogenous variable on itself and other variables in the system. Throughout the entire response period, land use efficiency exhibited a negative response (a) to positive shocks in population urbanization. As time progressed, this negative response gradually weakened, indicating that an increase in population urbanization in the short term would inhibit improved efficiency in land use. The efficiency of land use showed a positive response (b) to positive shocks in economic urbanization, with an initial increase during the first period, followed by a gradual decline. This suggests that an increase in economic urbanization in the short term can promote land use efficiency, but this promoting effect diminishes over time. Land use efficiency displayed a strong negative response (c) to positive shocks in social urbanization, which gradually weakened over time. This indicates that an increase in social urbanization in the short term would inhibit the improvement of land use efficiency. Land use efficiency exhibited a weak negative response (d) to positive shocks in ecological urbanization, with a further weakening of the negative response over time. This suggests that an increase in ecological urbanization in the short term only inhibits land use efficiency relatively weakly.

Throughout the entire response period, positive shocks in population urbanization elicited a positive response (e) in land use efficiency, peaking during the first period and subsequently gradually decreasing. This indicates that an improvement in land use efficiency in the short term encourages increased population urbanization. Positive shocks in economic urbanization led to a positive response (f) in land use efficiency, reaching its maximum during the first period and gradually declining. This suggests that an improvement in land use efficiency in the short term encourages increased economic urbanization. Positive shocks in social urbanization resulted in a positive response (g) in the efficiency of land use, with this positive response diminishing over time. This indicates that improved land use efficiency in the short term is beneficial for increasing the level of social urbanization. Positive shocks in ecological urbanization led to a positive response (h) in land use efficiency, reaching its maximum during the first period and gradually declining. This demonstrates that improved land use efficiency in the short term has a promoting effect on the level of ecological urbanization.

### 4.4 Discussion

In recent years, other researchers have also investigated correlations among urbanization and land use. Nevertheless, research conducted at the county-level scale within the Gansu section of the Yellow River Basin is somewhat scarce. There has been some exploration and analysis of the interplay and harmonious development between these two dimensions. For example, Wang and colleagues probed the state of coupling coordination between population urbanization and land urbanization within Gansu Province [44]. Xu and colleagues investigated the coupling coordination between the efficiency of the intensive use of land and the performance of prefecture-level cities in the Yellow River Basin in terms of their ecological welfare [45]. The analysis of the bidirectional interactive relationship between them has also been explored. Han and colleagues empirically analyzed the interactive effects of new urbanization and land-intensive utilization in the Beijing-Tianjin-Hebei conurbation [12]. Aimaiti and colleagues explored the reciprocal relationship between urbanization within the Wuhan city circle and land-intensive utilization [46]. These studies yielded findings akin to those presented in the present research.

This study still has some limitations. Firstly, in terms of the indicator system, some indicators could not be included due to difficult data acquisition, and in the future, the indicator system could be further refined and improved. Secondly, in the exploration of the interaction mechanisms between the two systems, the research was only conducted from the perspective of comprehensive land use efficiency, without categorizing land use types. In the future, in-depth research could be conducted from the perspective of different land use types.

In the course of promoting urbanization within the Gansu section of the Yellow River Basin, a reliance on ecological civilization is imperative, with a central focus on the development of ecologically sound land use practices. First, it is necessary to change the traditional development model, promote green transformation and further accelerate economic structural adjustment. Green development is the only way to promote the level of urbanization and land use efficiency, as well as to promote industrial transformation and upgrading through innovation. It is essential to adhere to the path of green transformation and integrate green development into the process of regional comprehensive development. Second, there should be a strong emphasis on technological innovation to foster the attraction of talents and knowledge, diminishing the reliance on traditional labor-intensive and resource-intensive industries. Third, when it comes to land development and utilization, it is crucial to take full account of regional characteristics, adopting strategies tailored to the unique conditions of different regional types.

## 5 Conclusions

The urbanization level and efficiency of land use in the Gansu section of the Yellow River Basin have exhibited a consistent upward trajectory, albeit at levels that are not particularly high, indicating substantial room for improvement in the future. Notably, the increase in urbanization level has slightly outpaced the growth in land use efficiency. Regarding spatial distribution patterns, both display some spatial disparities, albeit with a gradual reduction in regional inequalities. In general, urbanization in the Gansu section of the Yellow River Basin is characterized by low to moderate levels, whereas land use efficiency is generally considered inefficient.

The level of coupling coordination between urbanization and land use efficiency in the Gansu section of the Yellow River Basin has shown a generally upward trend. However, the overall coordination level remains relatively low, characterized by an imbalance, with “high coupling but low coordination”. Regarding spatial distribution patterns, considerable disparities exist in the level of coordination development, which generally decreases from the eastern toward the western regions. Specifically, the eastern region boasts relatively high-level coordination development, essentially reaching a “barely coordinated” status. The central region, centered around Lanzhou, also demonstrates a relatively high level of coordination development. By contrast, the western regions, including Gannan and Linxia Prefectures, generally exhibit an imbalanced status.

A strong reciprocal and interactive relationship exists between the urbanization level and land use efficiency. An elevated level of economic urbanization can initially stimulate land use efficiency, albeit with diminishing returns over time. Similarly, the improvement in the level of population urbanization, social urbanization, and ecological urbanization tends to exert a restraining influence on the augmentation of land use efficiency, although this inhibitory effect also gradually weakens. Conversely, the enhancement of land use efficiency makes a distinct contribution to promoting the elevation of the urbanization level, thereby contributing to the comprehensive enhancement of urbanization of all types: population, economic, social, and ecological.

## Supporting information

S1 Data (XLSX)
